# Ipconazole Disrupts Mitochondrial Homeostasis and Alters GABAergic Neuronal Development in Zebrafish

**DOI:** 10.3390/ijms24010496

**Published:** 2022-12-28

**Authors:** Giyoung Lee, Amit Banik, Juneyong Eum, Byung Joon Hwang, Seung-Hae Kwon, Yun Kee

**Affiliations:** 1 Department of Biomedical Science, College of Biomedical Science, Kangwon National University, Chuncheon 24341, Republic of Korea; 2 Interdisciplinary Graduate Program in Environmental and Biomedical Convergence, Kangwon National University, Chuncheon 24341, Republic of Korea; 3 Department of Molecular Bioscience, College of Biomedical Science, Kangwon National University, Chuncheon 24341, Republic of Korea; 4 Korea Basic Science Institute Seoul Center, Seoul 02841, Republic of Korea; 5 Division of Biomedical Convergence, College of Biomedical Science, Kangwon National University, Chuncheon 24341, Republic of Korea

**Keywords:** ipconazole pesticide, mitochondrial dysfunction, oxidative stress, neurodevelopmental toxicity, zebrafish

## Abstract

Ipconazole, a demethylation inhibitor of fungal ergosterol biosynthesis, is widely used in modern agriculture for foliar and seed treatment, and is authorized for use in livestock feed. Waste from ipconazole treatment enters rivers and groundwater through disposal and rain, posing potential toxicity to humans and other organisms. Its metabolites remain stable under standard hydrolysis conditions; however, their neurodevelopmental toxicity is unknown. We investigated the potential neurodevelopmental toxicity of ipconazole pesticides in zebrafish (*Danio rerio*). Our behavioral monitoring demonstrated that the locomotive activity of ipconazole-exposed zebrafish larvae was reduced during early development, even when morphological abnormalities were undetected. Molecular profiling demonstrated that the mitochondrial-specific antioxidants, superoxide dismutases 1 and 2, and the genes essential for mitochondrial genome maintenance and functions were specifically reduced in ipconazole-treated (0.02 μg/mL) embryos, suggesting underlying ipconazole-driven oxidative stress. Consistently, ipconazole treatment substantially reduced *hsp70* expression and increased ERK1/2 phosphorylation in a dose-dependent manner. Interrupted *gad1b* expression confirmed that GABAergic inhibitory neurons were dysregulated at 0.02 μg/mL ipconazole, whereas glutamatergic excitatory and dopaminergic neurons remained unaffected, resulting in an uncoordinated neural network. Additionally, ipconazole-treated (2 μg/mL) embryos exhibited caspase-independent cell death. This suggests that ipconazole has the potential to alter neurodevelopment by dysregulating mitochondrial homeostasis.

## 1. Introduction

The massive production and usage of pesticides and drugs introduce chemicals into the environment that may result in abnormal animal development, as well as neurological and psychiatric disorders. Therefore, tools must be developed to evaluate the potential intricate neurodevelopmental toxicities of these chemicals.

Ipconazole is a demethylation inhibitor of fungal ergosterol biosynthesis. It was developed as a fungicide in 1986 [1] and has since been widely used in Asia, Europe, North America, and Central and South America for foliar and seed treatments in modern agriculture. Additionally, according to the Organization for Economic Co-operation and Development (OECD) guidelines, ipconazole is authorized for use in cereals used to feed livestock [2]. Ipconazole is considered non-toxic to humans and animals [3]; therefore, the setting of maximum residue limits of it in commodities of animal origin has been deemed unnecessary. However, the neurodevelopmental toxicity of ipconazole remains untested.

Every year, a vast amount of chemical waste enters rivers, streams, and groundwater through rain or improper disposal. Risk assessment studies have suggested that residues of ipconazole were detected below 0.1 mg/kg in all commodities [4]. The total theoretical maximum daily intake was below 10% of the acceptable daily intake. Its derivatives remained stable under standard hydrolysis conditions [4].

Reactive oxygen species (ROS) serve as cell signaling molecules for normal biological processes, as well as for pathological events that cause damage to multiple cellular organelles and functions. When the cellular antioxidant defense system is disturbed, oxidative stress occurs, resulting in oxidative damage to nucleic acids, proteins, and lipids [5]. Mitochondria are the primary source of ROS, as well as one of the most sophisticated and dynamically responsive sensing systems in the body. Their dysfunction increases oxidative stress, and dysregulated mitochondrial genome and respiratory complexes are features of neurological disease pathogenesis and progression [6,7]. Thus, understanding the molecular mechanisms underlying mitochondrial dysfunction and oxidative stress will help to better define the acute toxicity of environmental chemicals, and establish a therapeutic strategy for addressing them.

Neurotransmitters are substances that neurons use to communicate with one another and their target tissues, and play a role in the excitatory or inhibitory effect of each specific neuron. Glutamate is the most potent excitatory neurotransmitter in the central nervous system. It ensures homeostasis along with *gamma-*aminobutyric acid (GABA), the most potent inhibitory neurotransmitter produced by neurons in the brain and spinal cord. Dopamine, a special type of neurotransmitter with both excitatory and inhibitory effects, inhibits unnecessary movement [8]. Disruption of neurotransmission alters its interactions in the neural network, which is associated with many neurological disorders, including depression, autism spectrum disorders, schizophrenia, Parkinson’s disease, and neurodegenerative diseases [9]. Therefore, assessing the neurodevelopmental toxicity of environmental chemicals, which drive neurodevelopmental defects and neurotransmission imbalances, is vital.

The zebrafish (*Danio rerio*) is a vertebrate model for 84% of human disease genes. Utilizing this practical and representative animal model, this study aimed to understand the molecular and cellular mechanisms underlying the potential developmental toxicity of ipconazole pesticides. We investigated the morphological and behavioral alterations in developing embryos treated with ipconazole, and elucidated the underlying molecular mechanisms. We believe that our study will provide a platform for understanding the molecular basis of neurodevelopmental toxicity derived from an environmental chemical.

## 2. Results

### 2.1. Ipconazole Pesticide Alters Larval Locomotive Behavior in Zebrafish Development

**Figure 1:**
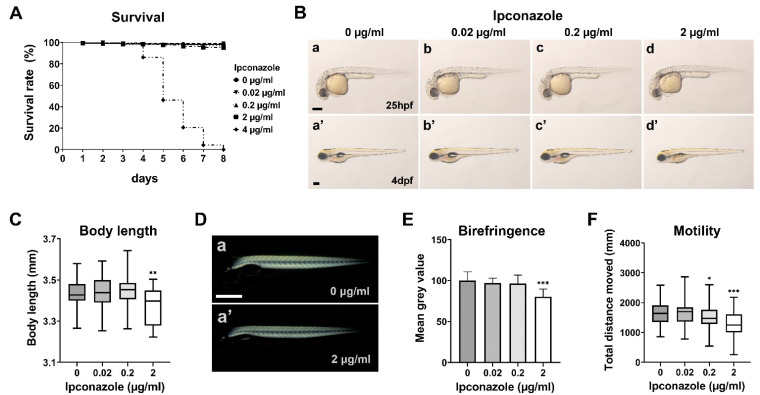
Ipconazole alters larval locomotive behavior in zebrafish development. (**A**) Survival rate (%) of zebrafish larvae treated with ipconazole, repeated six times (*n* = 300 each); (**B**) lateral views of the bright field imaging of the embryos at 25 h post fertilization (hpf) (**a**–**d**) and 4 days post fertilization (dpf) (**a’**–**d’**), repeated four times (*n* = 40 each), scale bar, 500 μm; (**C**) body length of the larvae treated with ipconazole at 4 dpf; (**D**) lateral views of 3-dpf larvae subjected to polarized light microscopy (**a**,**a’**), scale bar, 500 μm; (**E**) quantitation of the average birefringence of the larval muscle, repeated three times (*n* = 30 each); (**F**) locomotor activity of 6-dpf larvae treated with ipconazole. Graphical representation of the average distance traveled in the dark condition. * *p* < 0.05, ** *p* < 0.01, *** *p* < 0.001.

We examined the survival rate and morphological phenotypes of the embryos treated with ipconazole pesticide starting from the 50% epiboly stage. The larvae treated with 0.02, 0.2, or 2 μg/mL ipconazole survived until eight days post fertilization (dpf); however, all larvae were treated with 4 μg/mL ipconazole within 4–8 dpf (Figure 1A). Bright-field imaging showed no distinct morphological alteration of the embryo treated with 0.02 or 0.2 μg/mL at 25 h post fertilization (hpf) and 4 dpf (Figure 1B). The embryo treated with 2 μg/mL did not show abnormal morphology at 25 hpf; however, they had swim bladder defects (91.2% ± 13.31) (Figure 1A,B(d,d’)) and a shorter body length than the control group (98.3% ± 2.5) at 4 dpf (Figure 1C). Furthermore, the birefringence, representing muscle integrity, was maintained in the 3-dpf larvae treated with 0.02 and 0.2 μg/mL ipconazole and was significantly reduced in those treated with 2 μg/mL (Figure 1D,E). Notably, the locomotor activity of the larvae was significantly reduced at 0.2 μg/mL ipconazole (Figure 1F), even though morphological abnormalities were not observed (Figure 1B(c,c’)). These results suggest that ipconazole may have unobservable neurodevelopmental toxicity underlying abnormal behavioral activity during zebrafish development. 

### 2.2. Ipconazole Disrupts Mitochondrial Genome Maintenance and Functions

**Figure 2:**
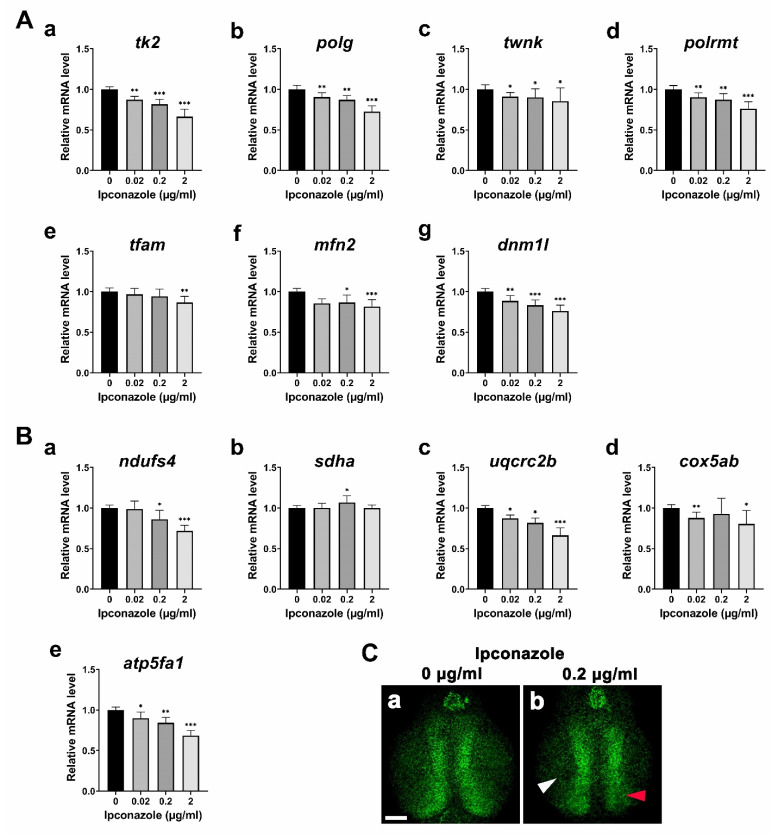
Ipconazole disrupts mitochondrial genome maintenance and respiratory complexes. (**A**) mRNA profiling of the genes involved in the mitochondrial DNA replication and transcription, *tk2* (**a**), *polg* (**b**), *twnk* (**c**), *polrmt* (**d**), and *tfam* (**e**), a mitochondrial fusion marker (*mfn2*) (**f**), and a fission marker (*dnm1l*) (**g**); (**B**) mRNA profiling of mitochondrial respiratory complex genes: *ndufs4* of Complex I (**a**), *sdha* of Complex II (**b**), *uqcrc2b* of Complex III (**c**), *cox5ab* of Complex IV (**d**), and *atp5fa1* of Complex V (**e**); (**C**) confocal images of mitochondria (in green) in the midbrain of 3-dpf *Tg(Xla.Eef1a1:mlsEGFP)^cms1^* larvae treated with ipconazole at 0 μg/mL (**a**), and 0.2 μg/mL (**b**). Scale bar, 50 μm; white arrowhead, neuropil; red arrowhead, periventricular zone. * *p* < 0.05, ** *p* < 0.01, *** *p* < 0.001.

To understand the molecular basis underlying the developmental toxicity of ipconazole pesticides, we first monitored alterations of the gene expressions involved in mitochondrial genome maintenance. The following genes involved in the replication and transcription of mitochondrial DNA (mtDNA) were significantly reduced in the embryo treated with 0.02 μg/mL ipconazole, and further in those treated with higher concentrations (Figure 2A(a–d)): *tk2* (*thymidine kinase-2*) for formation and maintenance of mtDNA, *polg* (*mtDNA polymerase gamma*) for mitochondrial genome replication, *twnk* (*twinkle mtDNA helicase*) for production and maintenance of mtDNA, and *polrmt* (*RNA polymerase mitochondrial*) for mitochondrial gene expression. In contrast, *tfam* (*mitochondrial transcription factor A*), an mtDNA transcription activator, was unaffected at 0.02 and 0.2 μg/mL but reduced at 2 μg/mL (Figure 2A(e)). According to the ipconazole-based disruption of mitochondrial DNA replication and transcription, we examined the alteration of genes involved in the fusion–fission cycle of mitochondria. A mitochondrial fusion marker, *mfn2* (*mitofusin 2*), and a fission marker, *dnm1l* (*dynamin-related protein 1*), were found to be significantly reduced in the embryos treated with 0.02 μg/mL ipconazole and further in those treated with higher concentrations (Figure 2A(f,g)). Furthermore, the expression of components of the mitochondrial respiratory complex was assessed (Figure 2B(a–e)). Particularly, *ndufs4* (*NADH–ubiquinone oxidoreductase iron–sulfur protein 4 subunit*) of mitochondrial respiratory Complex I was reduced in a concentration-dependent manner (Figure 2B(a)). However, *sdha* (*succinate dehydrogenase*) of Complex II was significantly increased only at 0.2 μg/mL, and *uqcrc2b* (*ubiquinol–cytochrome c reductase core protein II*) of Complex III was substantially reduced within the concentration range (Figure 2B(b,c)). Moreover, *cox5a*b (*cytochrome c oxidase subunit 5Ab)* of Complex IV was not considerably affected at 0.2 μg/mL but decreased at 0.02 and 2 μg/mL (Figure 2B(d)). Finally, *atp5fa1* (*ATP synthase mitochondrial F1 complex alpha subunit*) of Complex V was severely reduced in a dose-dependent manner (Figure 2B(e)). Confocal imaging revealed a decrease in the mitochondrial density in the forebrain of the 3-dpf larvae treated with ipconazole (Figure 2C(a,b)). These results suggest that ipconazole disrupts mitochondrial biogenesis and function during zebrafish embryonic development. 

### 2.3. Ipconazole Increases Oxidative Stress and ERK1/2 Activation

**Figure 3:**
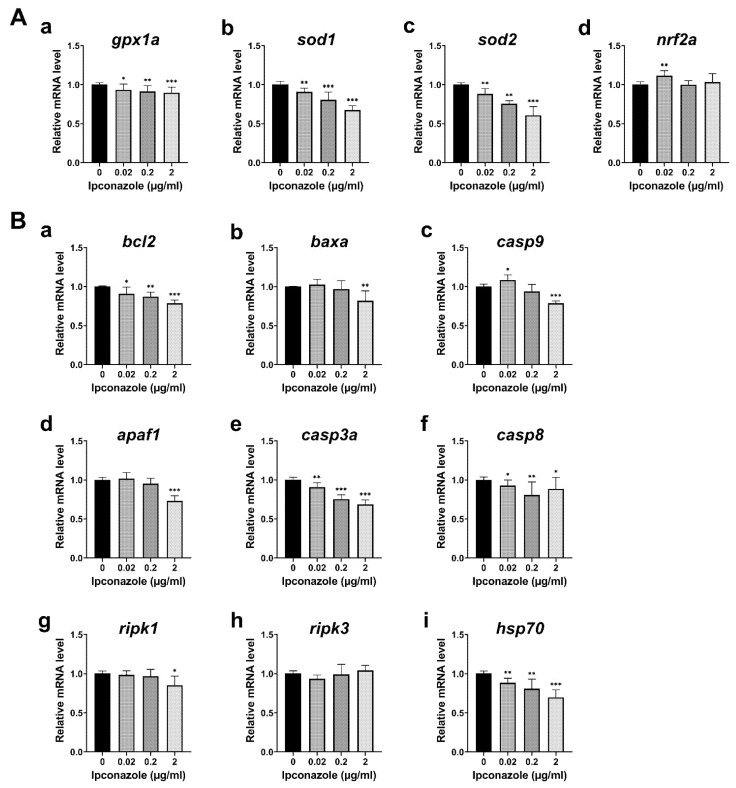
Ipconazole reduces expressions of antioxidant enzymes, caspases, and hsp70. (**A**) RT-PCR (real-time PCR) shows the antioxidant genes were reduced in the 28-hpf embryos treated with ipconazole at 0, 0.02, 0.2, and 2 μg/mL: *gpx1a* (**a**), *sod1* (**b**) *sod2* (**c**), and *nrf2* (**d**); (**B**) mRNA expression of the apoptotic genes, *bcl2* (**a**), *baxa* (**b**), *casp9* (**c**), *apaf1* (**d**), *casp3a* (**e**), and *casp8* (**f**), and the necroptotic genes, *ripk1* (**g**) and *ripk3* (**h**), as well as the molecular chaperon *hsp70* (**i**) were monitored. * *p* < 0.05, ** *p* < 0.01, *** *p* < 0.001.

Based on the mitochondrial defects in embryos exposed to ipconazole pesticide, we monitored the genes essential for the oxidative stress response in the developing embryo. The mRNA expression of antioxidant enzymes, *gpx1a* (*glutathione peroxidase 1*), *sod1* (*superoxide dismutase 1*), and *sod2,* were reduced in the 28-hpf embryos treated with ipconazole in a dose-dependent manner (Figure 3A(a–c)). The results indicate that the mitochondrial antioxidants *sod1*, *sod2*, and *gpx1a* are the most susceptible to ipconazole toxicity. The combined decrease in the levels of antioxidant enzymes may affect the vulnerability of developing zebrafish embryos to oxidative damage. Notably, *nrf2a* (*nuclear factor erythroid-2*a), a master coordinator of the antioxidant response [10], was upregulated at 0.02 μg/mL ipconazole (Figure 3A(d)), suggesting *nrf2a* may protect against ipconazole-induced oxidative stress at lower concentrations.

We then examined apoptotic regulation related to ipconazole-induced toxicity. Anti-apoptotic *bcl2* (BCL2 apoptosis regulator a), involved in mitochondrial-associated cell death, was significantly decreased in the 28-hpf embryos treated with ipconazole in a dose-dependent manner. In contrast, the proapoptotic *baxa* (*BCL2 associated X, apoptosis regulator a*) was slightly increased at 0.02 μg/mL and significantly reduced at 2 μg/mL (Figure 3B(a,b)). The expression of *casp9* (*caspase 9*), the key proapoptotic gene in the mitochondrial pathway, was significantly increased at 0.02 μg/mL. The expression of *apaf1* (*apoptotic peptidase activating factor 1*), an activator of mitochondrial apoptotic signaling, appeared to increase slightly (Figure 3B(c,d)). Expressions of *casp3a* (*caspase 3a*) and *casp8* (*caspase 8*) were decreased in a dose-dependent manner, and the necroptotic markers *ripk1* and *ripk3* (*receptor-interacting serine-threonine kinase 3*) were not significantly altered at 0.02 μg/mL, whereas *ripk1* (*receptor-interacting serine-threonine kinase 1*) was reduced at 2 μg/mL (Figure 3B(e–h)). In contrast, the expression of the molecular chaperone *hsp70* (*heat shock protein 70*) was significantly reduced in a dose-dependent manner (Figure 3B(i)). These results suggest that Hsp70-driven trafficking of mitochondrial membrane transporters and organismal homeostasis may be disrupted in embryos treated with ipconazole.

**Figure 4:**
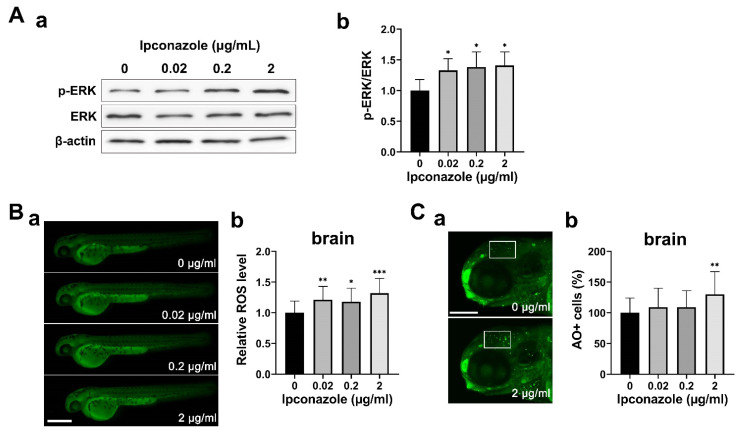
Ipconazole activates ERK1/2 signaling under oxidative stress. (**A**) Phosphorylation of ERK1/2 was increased in 28-hpf embryos treated with ipconazole. (**a**) Western blots using the antibodies against p-ERK, ERK, and β-actin proteins, respectively. (**b**) Graph of the quantitative analysis; (**B**) reactive oxygen species (ROS) were generated in the 48-hpf embryos treated with ipconazole. Scale bar, 500 µm. (**a**) Lateral views of the embryos subjected to DCFH-DA (2′,7′-dichlorodihydrofluorescein diacetate) staining and fluorescence imaging. (**b**) ROS level was quantitated by measuring the fluorescence intensity of ROS in the brain using the ImageJ software; (**C**) fluorescently labeled apoptotic cells in the brain (white box) of the 72-hpf ipconazole-treated embryos were counted using acridine orange staining. (**a**) Lateral views of the head subjected to acridine orange staining. Scale bar, 200 μm. (**b**) Graph of the quantitative analysis. * *p* < 0.05, ** *p* < 0.01, *** *p* < 0.001.

We further examined alterations in ERK1/2 (extracellular signal-regulated kinase 1/2) signaling. The ERK signaling system reportedly causes mitochondrial dysfunction in the presence of toxicity-induced stress [11]. A Western blot assay using anti-ERK1/2 and anti-p-ERK1/2 (phospho-ERK1/2) antibodies revealed a significant increase in ERK1/2 phosphorylation in ipconazole-treated embryos under ROS-induced oxidative stress (Figure 4A(a,b),B(a,b)). Moreover, cell death in the brain was significantly increased at 2 μg/mL, whereas a slight increase was observed in cell death at lower concentrations (Figure 4C). These results suggest that ipconazole disrupts mitochondrial homeostasis orchestrated by ERK1/2 activation, owing to underlying oxidative stress.

### 2.4. GABAergic Neuron Is Susceptible to Ipconazole Toxicity

**Figure 5:**
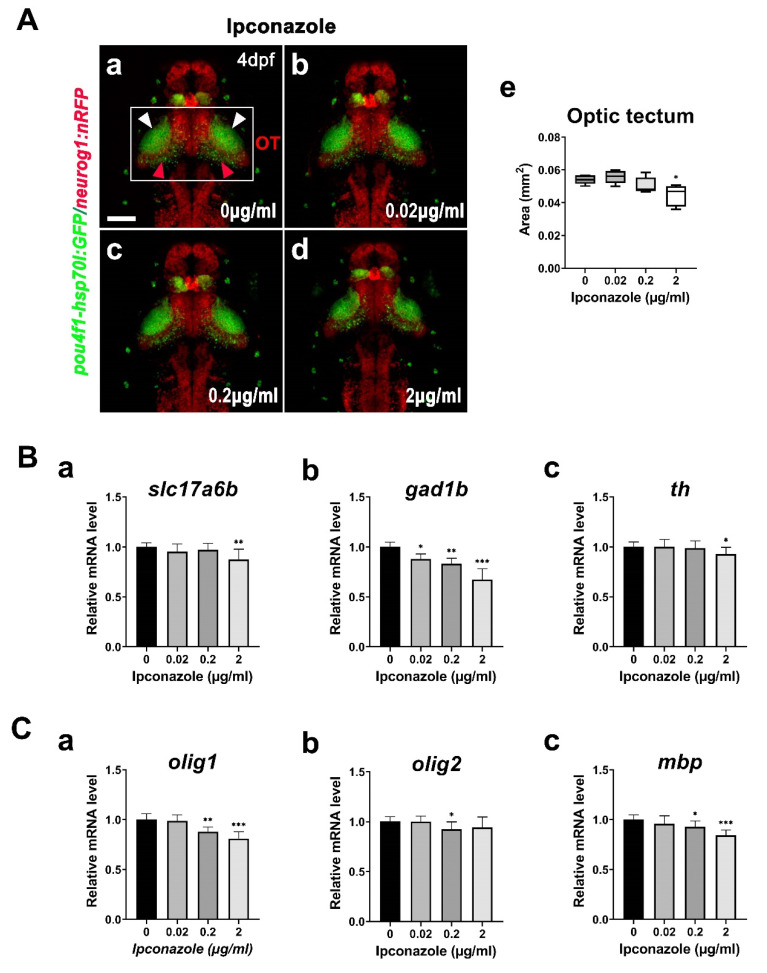
Ipconazole preferentially alters the development of GABAergic neurons. (**A**) Dorsal views of the brain of the 4-dpf larvae of *Tg(pou4f1-hsp70l: GFP)^rw0110b^* and *Tg(-8.4neurog1:nRFP)^sb3^* transgenic fish using confocal microscopy (**a**–**d**) and the size analysis of the optic tectum in the embryos treated with ipconazole (**e**). White box, optic tectum (OT); white arrowhead, neuropil; red arrowhead, periventricular layer, scale bar, 100 μm; (**B**) mRNA expressions of the following neuronal markers in the 3-dpf embryos using RT-PCR: *slc17a6b* for excitatory glutamatergic neurons (**a**), *gad1b* for GABAergic inhibitory neurons (**b**), and *th* for dopaminergic neurons (**c**); (**C**) mRNA expressions of the following glial cell markers: *olig1* and *olig2* for oligodendrocyte formation (**a**,**b**) and *mbp* for myelination (**c**) in the 3-dpf embryos. * *p* < 0.05, ** *p* < 0.01, *** *p* < 0.001.

To visualize the anatomical alteration underlying the neurodevelopmental toxicity of ipconazole in the zebrafish brain, we used the *Tg(-8.4neurog1:nRFP)^sb3^/(pou4f1-hsp70l:GFP)^rw0110b^* double line for imaging the whole brain and the midbrain structures, including the tectal neuropil and periventricular layer, using confocal microscopy (Figure 5A(a–d)). The size of the optic tectum in the brain was significantly reduced by 2 μg/mL ipconazole only (Figure 5A(e)).

We next monitored the molecular changes in the following gene expressions during the development of excitatory neurons, inhibitory neurons, and dopaminergic neurons in the brain of 3-dpf larvae treated with ipconazole: *slc17a6b* (*solute carrier family 17 member 6b*) for glutamatergic excitatory neurons, *gad1b* (*glutamate decarboxylase 1b*) for GABAergic inhibitory neurons, and *th* (*tyrosine hydroxylase*) for dopaminergic neurons. Notably, the *gad1b* expression was specifically reduced in the 3-dpf larvae treated with 0.02 μg/mL ipconazole, whereas *slc17a6b* and *th* expressions were unaffected (Figure 5B(a–c)). The results indicated that GABAergic neurons in the brain were more susceptible to ipconazole toxicity than glutamatergic and dopaminergic neurons, resulting in neurotransmission imbalance. We also measured the mRNA expression levels of the following genes involved in the development of glial cells: *olig1* (*oligodendrocyte transcription factor 1*) and *olig2* (*oligodendrocyte transcription factor 2*) in oligodendrocyte precursors and *mbp* (*myelin basic protein a*) in myelination. None of the glial genes were affected at 0.02 μg/mL ipconazole; however, *olig1* and *mbp* expression were significantly reduced at 0.2 and 2 μg/mL (Figure 5C). These results demonstrated that ipconazole differentially regulates neuronal and glial cell populations in the developing nervous system.

## 3. Discussion

Our behavioral monitoring was sensitive enough to reveal the reduced locomotive activity of ipconazole-treated zebrafish larvae, even when morphological abnormalities were not detected by conventional microscopy. This result paved the way for a further comprehensive assessment of its neurodevelopmental toxicity at the molecular and cellular levels. 

To understand the molecular mechanism of the undetectable toxicity underlying ipconazole-derived developmental alterations, we profiled the expression of various genes using RT-PCR. It has been reported that *sod2* knockout mice die within 3 weeks of birth due to cardiomyopathy, metabolic acidosis, and neurodegeneration [12]. In the current study, mitochondrial *sod1* and *sod2* were significantly reduced in developing embryos treated with ipconazole, whereas ROS were generated, indicating the vulnerability of mitochondrial antioxidants to oxidative stress. Mitochondrial gene expression profiling indicated the adverse effects of ipconazole on mitochondrial biogenesis and function in zebrafish embryonic development. Notably, Hsp70 is essential for the trafficking of membrane transporters into mitochondria. Transporters of the mitochondrial inner membrane are synthesized as nascent precursors in the cytoplasm, bind to the Hsp70 and Hsp90 chaperones, and are subsequently delivered to the mitochondria [13]. The ipconazole-driven decrease in *hsp70* expression may disrupt this trafficking of mitochondrial membrane transporters. Overall, the combined data suggest that ipconazole disrupts mitochondrial homeostasis underlying oxidative stress.

The ERK is a crucial signaling pathway that regulates cell growth, differentiation, apoptosis, and homeostasis at multiple levels. However, under abnormal circumstances, activated ERK leads to various pathological changes [14,15]. Additionally, ROS can promote persistent activation and nuclear accumulation of ERK1/2, which is associated with apoptosis [16,17]. Furthermore, it has been shown that ERK1/2 activation facilitates neuronal cell death without caspase activation [18]. Our findings suggest that neuronal cell death, caused by high-dose (2 μg/mL) ipconazole treatment, likely results from ROS-induced ERK1/2 activation, a caspase-independent process.

**Figure 6:**
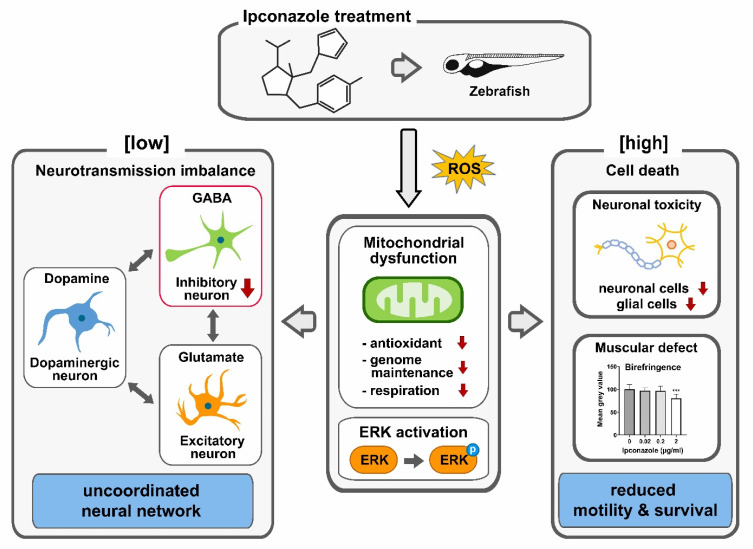
Potential mechanism of neurodevelopmental toxicity of ipconazole pesticide in zebrafish. The zebrafish embryos treated with ipconazole generate ROS and show evidence of dose-dependent mitochondrial dysfunction and ERK1/2 activation. Ipconazole specifically inhibited the development of GABAergic inhibitory neurons at low doses. In contrast, glutamatergic and dopaminergic neurons remained unaffected, possibly disrupting the interplay among the major neurotransmission systems and uncoordinated neural functions in the central nervous system. High concentrations of ipconazole cause cell death in neuronal and muscle development, reducing the motility and survival of embryos. *** *p* < 0.001.

Our molecular analyses demonstrate that the development of *gad1b*-positive GABAergic inhibitory neurons is the most susceptible to ipconazole exposure at 0.02 μg/mL, whereas *slc17a6b*-expressing glutamatergic excitatory neurons and *th*-expressing dopaminergic neurons remain unaffected. The differential interplay between these three different types of neurons plays a vital role in the coordinated neuronal communication within the central nervous system [19]. The present data implicate GABAergic inhibitory neurons as the primary targets of ipconazole exposure among the major neurotransmission systems in the developing brain, possibly resulting in the development of an uncoordinated neural network with neurotransmission imbalance (Figure 6). The potential developmental toxicity of ipconazole is summarized in Figure 6.

Dechorionated embryos may be more sensitive to evaluating environmental chemicals at low concentrations without a barrier [20,21]. However, the physical dechorionation of embryos is labor intensive, and enzymatic dechorionation introduces additional stress and variation to developing embryos; thus, we used membrane-intact embryos in this study. Different environmental chemicals may initiate unique molecular signaling events, resulting in differential developmental toxicities in animals, depending on their concentration range and stability. Therefore, it would be beneficial to develop a high-throughput screening method utilizing the zebrafish assessment systems discussed in the present study to screen potentially harmful chemicals for neurodevelopmental toxicity.

## 4. Materials and Methods

### 4.1. Zebrafish Genetic Background and Maintenance

Wild-type AB and AB* zebrafish *(Danio rerio)* were purchased from the Zebrafish International Resource Center (Eugene, OR, USA). The transgenic fluorescence reporter strains used in this study visualized the following specific tissues: *Tg(-8.4neurog1:nRFP)^sb3^* (ZDB-ALT-030904-8) labeled neuronal progenitors in the brain in red [22], *Tg(Xla.Eef1a1:mlsEGFP)^cms1^* (ZDB-ALT-090309-2) marked mitochondria in green [23], and *Tg(pou4f1-hsp70l:GFP)^rw0110b^* fluorescently labeled the dorsal habenula of the midbrain in green as well [24,25]. Adult zebrafish were raised in balanced saltwater at 27.5 °C on a 14 h/10 h light/dark cycle. The developmental stages of the embryos were recorded as hpf and dpf [26]. All procedures were approved by the Animal Use and Ethics Committee of Kangwon National University (Chuncheon, Republic of Korea) and were performed in accordance with the IACUC principles and the National Law for Laboratory Animal Research.

### 4.2. Chemical Treatment

The chemicals used in this study were of analytical grade unless otherwise described. Embryos were obtained through natural spawning, raised at 28.5 °C in E3 medium (5 mM NaCl, 0.33 mM MgSO_4_, 0.33 mM CaCl_2_, and 0.17 mM KCl). Ipconazole pesticide (8% ipconazole) was purchased from Farm Hannong Co., Ltd. (Seoul, Republic of Korea) and dissolved in E3 medium. Embryos were treated from the 50% epiboly stage with ipconazole at the concentrations of 0, 0.02, 0.2, 2, and 4 μg/mL in a 35 mm petri dish and incubated at 28.5 °C until examined.

### 4.3. Bright-Field Imaging

Treated embryos were examined for survival rates at 24, 48, 72, and 96 hpf using an Olympus stereoscope (Olympus, Tokyo, Japan). Each embryo was anesthetized with 0.02% tricaine (Sigma-Aldrich Corp., St. Louis, MO, USA) in an E3 medium, mounted with 3% methylcellulose (Sigma-Aldrich Corp., St. Louis, MO, USA). The bright field images were captured using an Olympus stereoscope (Olympus, Tokyo, Japan) with an AxioCam GRC camera (Carl Zeiss, Overkochen, Germany) using the Zeiss Zen 3.4 (Blue edition) software [27]. The body length and brain size of the embryos were measured using polygon selection in ImageJ software (http://rsbweb.nih.gov/ij/, accessed on 28 December 2021). All experiments were repeated at least thrice. 

### 4.4. Birefringence Measurement

For quantitative birefringence measurements, we employed a previously described method [28,29]. First, a single polarizing filter was applied to all images even though they were taken under similar circumstances. On one microscope slide, 3% methylcellulose was placed, along with a sedated (0.02% tricaine) 3 dpf-stage larva. The microscope slide containing the larva was then placed at the correct spot on a fixed polarizing filter. Subsequently, another polarizing filter was placed at a 90-degree angle across the crossed polarizers to obtain the brightness of the muscle tissue. Images were captured using Olympus ZEN microscope software (Tokyo, Japan). The trunk musculature was traced using ImageJ’s polygon selection tool (http://rsbweb.nih.gov/ij/, accessed on 1 May 2021), and the average mean gray value inside the resulting selection was obtained.

### 4.5. Locomotive Behavior Test

At 5 dpf, each larva was individually transferred to a well of a 96-well plate with 200 µL of E3 medium and incubated until 6 dpf. The 96-well plate was set inside a DanioVision observation chamber (Noldus, Wageningen, The Netherlands). The locomotor activity of 6 dpf zebrafish larvae was monitored in the following light–dark transition cycle using the high-quality video tracking system at 28.5 °C: a 10-min dark acclimation period was followed by alternating 10 min light and dark cycles for 60 min. EthoVision XT14 software (Noldus, Wageningen, The Netherlands) was used to trace and quantitatively analyze larval locomotor activity. The locomotor activity of each larva was measured as the average distance traveled during a light–dark transition cycle (mm). All experiments were repeated at least three times.

### 4.6. Real-Time PCR (RT-PCR)

Total RNA was isolated from 20 embryos per exposure group at 28 hpf and 20 larvae per exposure group at 72 hpf. Then, cDNA was generated using the PrimeScript™ first strand cDNA Synthesis Kit according to the manufacturer’s instructions (TAKARA, Republic of Korea). Finally, RT-PCR was performed using SYBR Green with low ROX (TOPreal™ qPCR 2X PreMIX, Cat-RT500M, Republic of Korea) in the Applied Biosystems Real-Time PCR System (QuantStudio 1, Applied Biosystems, Thermo Fischer Scientific). The amplification conditions were 95 °C for 10 min, 40 cycles of 95 °C for 10 s, 60 °C for 15 s, and 72 °C for 15 s, followed by the conditions necessary for the calculation of the melting curve (95 °C for 15 s, 60 °C for 1 min, and 95 °C for 0.1 s). Each sample was tested in triplicate, with β-actin as the control on each plate. The transcription levels of genes involved in oxidation (4 genes), mitochondrial physiology (12 genes), cell death (9 genes), neural (3 genes), and glial (3 genes) development were normalized in relation to the β-actin gene. The mRNA transcripts were quantified using the 2^−ΔΔCt^ method. The RT-PCR primers used are listed in Supplementary Table S1.

### 4.7. Western Blot Analysis

Ipconazole-treated 3-dpf larvae were homogenized with a plastic pestle in 1x RIPA buffer (IBS-BR004, iNtRON, Seongnam, Republic of Korea) containing 1x Xpert duo inhibitor cocktail solution (P3300, GenDEPOT, Baker, TX, USA) and centrifuged. The supernatant containing total protein was prepared as an SDS sample buffer. Samples were electrophoresed on 13% SDS-PAGE gel and transferred to a polyvinylidene difluoride (PVDF) membrane (Merck, Darmstadt, Germany). Membranes were blocked at 23 °C with 5% skim milk/TBST or 5% BSA/TBST and incubated overnight at 4 °C with primary antibodies: rabbit monoclonal anti ERK1/2 (CST #4370, Cell Signaling Technology, Beverly, MA, USA) in 5% skim milk/TBST (1:1000); rabbit monoclonal phospho-ERK1/2 (137F5) (CST #4695, Cell Signaling Technology, MA, USA) in 5% BSA/TBST (1:2000); mouse monoclonal anti-β-actin (sc-47778, Santa Cruz Biotechnology, Dallas, TX, USA) in 5% skim milk/TBST (1:2500). The secondary antibodies used were HRP-conjugated anti-rabbit (GenDEPOT, SA002) (1:2000) or HRP-conjugated goat anti-mouse (SA001, GenDEPOT) (1:20,000). Protein bands were imaged using a chemiluminescence substrate kit (W365100-012, GenDEPOT) and the ImageQuant LAS 500 imaging system (General Electric Healthcare, Chicago, IL, USA), and relative quantification was performed using ImageJ 1.53q (U. S. National Institutes of Health, Bethesda, MD, USA).

### 4.8. Measurement of Reactive Oxygen Species (ROS)

To quantify ROS production in zebrafish embryos, 2′,7′-dichlorodihydrofluorescein diacetate (DCFH-DA) (D6883, Sigma-Aldrich Corp., St. Louis, MO, USA) was used. At 2 dpf, embryos treated with ipconazole were dechorionated using forceps and transferred into each well of a 9-well plate. After removing the E3 medium, each embryo was treated with 200 μL of 1 μg DCFH-DA/0.5% DMSO/E3 medium and incubated for 1 h in dark conditions at 28.5°. ROS production was visualized using green fluorescence, detected with an Olympus SZX16 stereoscope (Olympus, Tokyo, Japan) equipped with a mercury light source (U-LH100HGAPO, Olympus, Tokyo, Japan) stereo microscope, and AxioCam HRc camera (Carl Zeiss, Oberkochen, Germany), and was quantitatively analyzed using ImageJ.

### 4.9. Acridine Orange Staining

Acridine orange (A6014, Sigma-Aldrich, St. Louis, MO, USA) dye was used to observe apoptotic cell death in living larvae. At 3 dpf, larvae were stained for 20 min in 3 μg/mL acridine orange in E3 medium under dark conditions at room temperature and then washed three times with E3 medium for 5 min each. Washed larvae were anesthetized with 0.02% tricaine and then mounted in a 35 mm confocal dish with 0.8% low melting point agarose. Imaging apoptotic cells in living larvae was performed using a Nikon Eclipse Ti2 confocal microscope (488 nm Ar-laser, 520-535 BP filter, Ts2, NIKON, Tokyo, Japan).

### 4.10. Confocal Microscopy of Transgenic Larvae

The brains of 4 dpf larvae of the transgenic fluorescence reporter lines, *Tg(Xla.Eef1a1:mlsEGFP)^cms1^*, *Tg(-8.4neurog1:nRFP)^sb3^*, and *Tg(pou4f1-hsp70l:GFP)^rw0110b^* were mounted as previously described [30]. Fluorescent images were collected using a Nikon Eclipse Ti2 confocal microscope with Lambda D series objectives (10×, 20×, 40×, 60×) (Ts2, NIKON, Tokyo, Japan) in the Kangwon Center for System Imaging (KCSI, Chuncheon, South Korea). GFP/RFP was observed using a 488 nm Ar laser (520–535 BP filter)/568 nm HeNe-laser (LP 596 filter). The maximum intensity projection was used to merge the optical sections for each z-stack. The stacked sections were merged for each region of interest to create an image, and NIS-Elements AR 5.11.00 64-bit (NIKON, Tokyo, Japan) software was used to process the raw data from confocal imaging. 

### 4.11. Statistical Analysis

Statistical analyses were performed, and graphs were generated using GraphPad Prism (v.9; GraphPad Software, San Diego, CA, USA). All quantified values represent mean ± standard deviation (SD) per embryo or phenotype of at least three independent experiments. Differences between the control and treated groups were evaluated using one-way ANOVA, followed by Student’s t-tests. Significance was set at *p* < * 0.05, ** 0.01, and *** 0.001.

## 5. Conclusions

Our findings demonstrated that ipconazole pesticide disrupts mitochondrial homeostasis, and particularly pinpointed the GABAergic inhibitory neuron as the primary target among the major categories of neurons in the central nervous system. The present assessment, including behavioral monitoring as the primary developmental toxicity assessment in addition to basic anatomical observation, provides a robust platform for identifying potentially toxic environmental chemicals, and furthering our understanding of the molecular basis underlying neurodevelopmental toxicity.

## Data Availability

Not applicable.

## Supplementary Materials

The supporting information can be downloaded at: https://www.mdpi.com/article/10.3390/ijms24010496/s1.
