# Supplementary material for: Ipconazole Disrupts Mitochondrial Homeostasis and Alters GABAergic Neuronal Development in Zebrafish
Source: Int J Mol Sci. 2022 Dec 28;24(1):496. doi: 10.3390/ijms24010496 (PMC9820214; doi:10.3390/ijms24010496)
Supplement: Supplementary file 1 [file ijms-24-00496-s001.zip › ijms-2089467-supplementary.pdf]

Ipconazole Disrupts Mitochondrial Homeostasis and Alters GABAergic Neuronal Development in Zebrafish

Giyoung Lee [1,†], Amit Banik [2,†], Juneyong Eum [2], Byung Joon Hwang [3], Seung-Hae Kwon [4,*] and Yun Kee [5,*]

[1] Department of Biomedical Science, College of Biomedical Science, Kangwon National University, Chuncheon 24341, Republic of Korea
[2] Interdisciplinary Graduate Program in Environmental and Biomedical Convergence, Kangwon National University, Chuncheon 24341, Republic of Korea
[3] Department of Molecular Bioscience, College of Biomedical Science, Kangwon National University, Chuncheon 24341, Republic of Korea
[4] Korea Basic Science Institute Seoul Center, Seoul 02841, Republic of Korea
[5] Division of Biomedical Convergence, College of Biomedical Science, Kangwon National University, Chuncheon 24341, Republic of Korea
* Correspondence: kwonsh@kbsi.re.kr (S.-H.K.); yunkee@kangwon.ac.kr (Y.K.)
† These authors contributed equally to this work.

Table S1. RT-PCR primers

Types	Official Gene name	Forward primer	Reverse primer	Zebrafish mRNA ID
**Control**	**β-actin**	GTGATGGTTGGCATGGGACAG	CCAGTTGGTCACAATACCGTGC	NM_131031.2
**Mitochondria genome maintenance**	**tk2**	CCTGTATGAGGACTGGCTGA	TCTGTTCTCCTCAAACTGATGC	XM_017356845.2
	**polg**	GGTGACCAGTGAAGACCGATA	GTCCACTGCGCTAAAGAAGG	XM_001921095.6
	**twnk**	TGTGGGCTGACAAGTTTGAGG	TGTCCACAGACAGATTTTCTTG	NM_001277598.2
	**polrmt**	ACCCGCTGCCGCCTTATTTT	TCCAGCGAGCTCTGCTTCTTC	XM_005170522.3
	**tfam**	GCGAAAGATTGCCCAGCAGT	TTGTCGTTTTTCCTCCGCAAA	NM_001077389.1
**Mitochondria fusion & fission**	**mfn2**	GGCAGGACCAGTAACGGAAA	TTTCGTCCGACCCTTCAGTG	XM_005172484.4
	**dnm1l**	CGCAGAGTAGCGGGAAGAG	CATCCACTCCGTTCTCCTCG	XM_005163049.2
**Mitochondria respiratory complexes I-V**	**ndufs4**	TGTAGGCTGGCAGAGGGACA	GACAGGCCGAAACAGGATGG	NM_001020676.1
	**sdha**	TGGTATGCCGTTCAGCCGTA	GGCCAAGTCTTTGGCATTGG	NM_200910.1
	**uqcrc2b**	GACCTCACGGGAAGGGTGAA	TCAGTGTGCTGGTGCTGCTG	NM_001001589.1

	**cox5ab**	GGTCACCGGAGCTTCAGGAT	TCGAGCCGAGAGGTAGAAAAACC	NM_001305577.1
	**atp5fa1**	TTCTTGGAGCCGACACTGGA	CGAACACCACAACACCAACG	NM_001077355.1
**Oxidative stress**	**gpx1a**	AGATGTCATTCCTGCACACG	AAGGAGAAGCTTCCTCAGCC	NM_001007281.2
	**sod1**	GGGCCAACCGATAGTGTGAG	ACCCTTCCCCAAGTCATCCT	NM_131294.1
	**sod2**	CCGGACTATGTTAAAGCCATCT	ACACTCGGTTGCTCTCTTTTCTCT	NM_199976.1
	**nrf2a**	ATGTCTAAAATGCAGCCAAGCC	CGGTAGCTGAAGTCGAACAC	XM_005172511.4
**Apoptotic marker**	**bcl2a**	AGGAAAATGGAGGTTGGGATG	TGTTAGGTATGAAAACGGGTGGA	NM_001030253.2
	**baxa**	GGCTATTTCAACCAGGGTTCC	TGCGAATCACCAATGCTGT	NM_131562.2
	**casp9**	AAATACAGAGCAAGGCAACC	CACAGGGAATCAAGAAAGG	NM_001007404.2
	**apaf1**	TTCTACAGTAAACGCCCACC	TATCTAGTATTTCCCCATATTCC	XM_021475139.1
	**casp3a**	CCGCTGCCCATCACTA	ATCCTTTCACGACCATCT	NM_131877.3
	**casp8**	GGAATGATCTGGAAGCCTGGG	TCCGGCAAAAGGCAGTGTAA	NM_131510.2
**Necroptotic marker**	**ripk1**	TCCTGGACCAAACCATCAGC	ACTGACTCCTCAATTCGGGC	NM_001043350.1
	**ripk3**	AGCAAAGCCTTCGACGTGTA	CATCCAGTCCTTCGGTCTCG	XM_001343791.5
**Heat shock protein marker**	**hsp70**	GCACAAGAAGGACATCAGTCAGA	CGATGCCCTCGTACAGAGAGT	NM_001362359.1
**Neurotransmitter marker**	**slc17a6b**	GAACTGACCGAGGATGGACG	TTGCTTTTATGGCCACGCCT	NM_001005398.1
	**gad1b**	GTTCAGCCATCCTGGTCAGA	CCTTGTCTCCGGTGTCGTAG	XM_009302104.3
	**th**	GACGGAAGATGATCGGAGACA	CCGCCATGTTCCGATTTCT	NM_131149.1
**oligodendrocytes/ myelin marker**	**olig1**	CGGACTGAAAGTTTGAAGAATGC	TCCTGTTACCCGTACCATTCTTG	XM_005167653.4
	**olig2**	ATCCGTCCAGTTGTGGCACT	TGGTGGAAGCAGAGGATGGT	NM_178100.1
	**mbp**	CCGTCGTGGAGACGTCAA	CGAGGAGAGGACACAAAGCT	XM_021468312.1
